# Evaluation of Ni-Based Flexible Resistance Temperature Detectors Fabricated by Laser Digital Pattering

**DOI:** 10.3390/nano11030576

**Published:** 2021-02-25

**Authors:** Vu Binh Nam, Daeho Lee

**Affiliations:** Laser and Thermal Engineering Lab, Department of Mechanical Engineering, Gachon University, Seongnam 13120, Korea; vubinhnam@gmail.com

**Keywords:** laser digital patterning, NiO*_x_* nanoparticle ink, laser-induced reductive sintering, Ni electrodes, flexible resistance temperature detector

## Abstract

Temperature sensors are ubiquitous in every field of engineering application since temperature control is vital in operating, testing and monitoring various equipment systems. Herein, we introduce a facile and rapid laser digital patterning (LDP) process to fabricate low-cost, Ni-based flexible resistance temperature detectors (RTDs). Ni-based RTDs are directly generated on a thin flexible polyimide substrate (thickness: 50 µm) by laser-induced reductive sintering of a solution-processed nonstoichiometric nickel oxide (NiO*_x_*) nanoparticle thin film under ambient conditions. The shape of RTDs can be easily adjusted by controlling computer-aided design (CAD) data without using the physical patterning mask while the sensitivity (temperature coefficient of resistance (α) ~ 3.52 × 10^−3^ °C^−1^) of the sensors can be maintained regardless of shape and size of the sensor electrodes. The flexible Ni-based RTDs can operate over a wide temperature range up to 200 °C with excellent repeatability. Additionally, the Ni-based RTDs respond quickly to the temperature change and can operate in corrosive environments including water and seawater. Moreover, the Ni-based RTDs show a superior mechanical and electrical stability with a negligible resistance change up to a radius of curvature of 1.75 mm. Finally, a tape-pull test demonstrates the robust adhesion of Ni-based RTDs on the substrate.

## 1. Introduction

Resistance temperature detectors (RTDs) are extensively adopted as temperature sensors in every field of industrial, consumer, automotive, and medical electronics applications because temperature control and monitoring are vital. The basic principle of RTDs is an increase in electrical resistance of metallic electrodes upon temperature change with a positive temperature coefficient (PTC). Various conventional vacuum-based deposition methods such as sputtering [1,2], chemical vapor deposition (CVD) [3,4], electron beam evaporation [5,6,7], and thermal evaporator [8,9] have been extensively employed to fabricate RTDs. However, the fabrication of flexible RTDs faces a major challenge for actual utilization since the required high temperature in these fabrication processes can destroy the flexible polymer substrates. On the other hand, the need for customizable sensors is increasing due to the emergence of wearable electronics, artificial skins, health monitoring kits, and soft robotics [10,11,12,13,14,15,16,17,18,19] that require more complicated designs and seamless integration between components. To meet these requirements, a new fabrication process to fabricate lightweight and flexible RTDs should be developed, which enables direct deposition of sensor electrodes onto heat-sensitive polymer substrates as well as altering of sensor design in a simple way.

Laser digital patterning (LDP) process of solution-processed thin films has recently emerged as a suitable tool to realize electrode patterning on flexible substrates due to its low thermal stress applied to the substrate [20,21,22,23,24,25,26,27,28,29,30,31,32]. In the process, no vacuum-based equipment is necessary because thin films can be deposited by spin-coating or blading of nanoparticle (NP) or composite ink. Especially, the pattern designs in the LDP process can be easily controlled simply by changing the computer-aided design (CAD) data instead of altering a physical patterning mask. Au and Ag NPs have been extensively utilized in the early stage of the LDP process because such noble materials are not easily oxidized even in nanoscale, and are easily fused or sintered together to form continuous conductive structures upon laser irradiation owing to their low melting temperature [33,34,35]. Even though Au [36,37] and Ag [38,39,40] have been widely employed for the temperature sensing applications, the high price of the noble metals presents a major challenge for practical usage. Hence, using non-noble metals for the LDP process is becoming increasingly important. However, non-noble metal NPs are easily oxidized and exist as metal oxides unless special treatment is applied. Therefore, to form non-noble metal electrodes by the LDP process, metal oxide NPs should be reduced and sintered simultaneously, which is referred to as so-called the laser-induced reductive sintering (LRS) phenomenon [41,42,43]. Hata et al. reported the fabrication of Cu-based RTDs by reductive sintering of CuO NPs using femtosecond laser pulses [44]. They also demonstrated that the resistance of the laser-irradiated Cu electrodes displayed a metal-like electrical conductivity behavior with a PTC whereas the resistance of Cu_2_O electrodes showed a semiconductor-like behavior with a negative temperature coefficient (NTC), which could be achieved by controlling the reduction degree of CuO NP thin films during the LRS phenomenon [45]. However, it remains a challenge to use Cu electrodes as temperature sensors because Cu is prone to oxidation even under ambient conditions leading to performance fluctuation.

In contrast to Cu, Ni has high thermal, chemical stability with silver-like color, thus is used as a component of corrosion-resistant alloys [46,47,48]. In addition, bulk Ni possesses a relatively higher temperature coefficient of resistance (*α*) (*α*_Ni_ = 5.866 × 10^−3^ °C^−1^) compared to Cu (*α*_Cu_ = 4.041 × 10^−3^ °C^−1^) and other noble metals (*α*_Au_ = 3.715 × 10^−3^ °C^−1^, *α*_Ag_ = 3.819 × 10^−3^ °C^−1^) [49]. For these reasons, Ni has been widely utilized for temperature sensors [50,51,52]. Atashbar et al. reported the screen printing process to fabricate a Ni-based RTD [50]; Bao et al. demonstrated a Ni-filled binary polymer composite temperature sensor with high thermal cycling stability and tunable temperature range [51]; Yuan et al. developed Ni flexible thermal sensor arrays for underwater applications [52]. However, the limitations such as low thermal stability [51], vacuum-based thin film deposition [52], insufficient adhesion strength of the sensors on the substrate [50], and fixed design patterns [50,51,52] have room for improvement.

In this study, we introduce a facile and rapid method to fabricate Ni-based flexible RTDs by the LDP process of solution-processed NiO*_x_* NP thin films. Entire processes covering from synthesis of NiO*_x_* NPs to the laser process are conducted under ambient conditions. Continuous-wave (CW) laser beam irradiation on the selected areas of the NiO*_x_* thin film coated on very thin (≤50 µm) flexible substrates induces the reductive sintering phenomenon to generate Ni electrode patterns that are used for RTDs. Various shapes and sizes of Ni-based RTDs can be facilely produced without using physical photomasks. Although the shapes of RTDs are varied, the temperature coefficient of resistance, or sensitivity of the RTDs is maintained, which is advantageous in practical usage by offering design flexibility. The flexible Ni-based RTDs can operate over a wide temperature range up to 200 °C with fast response and excellent repeatability. Moreover, Ni-based RTDs can detect very small temperature variations (the temperature of the gloved finger) and can operate normally in corrosive environments such as water and seawater. The superior mechanical and electrical stability of the RTD on PI is confirmed through the bending and tape-pull tests.

## 2. Materials and Methods

### 2.1. Synthesis of NiO_x_ NPs Ink and NiO_x_ Thin Film Deposition

NiO*_x_* NPs were prepared following the chemical precipitation method reported in previous literature with several modifications [53]. Nickel(II) nitrate hexahydrate (Ni(NO_3_)_2_·6H_2_O), polyvinylpyrrolidone (PVP, molecular weight ≈ 10,000), sodium hydroxide (NaOH), and 1-pentanol were supplied from Sigma-Aldrich, St. Louis, MO, USA. Ni(NO_3_)_2_·6H_2_O (0.05 mol) was dispersed in 100 mL of deionized (DI) water. After the pH of the solution was controlled to 10 by dropwise adding of NaOH solution (10 M), the green nickel hydroxide (Ni(OH)_2_) was produced as a colloidal suspension. The colloidal precipitate was separated from the liquid phase by centrifuging at 3000 rpm for 5 min, and the upper liquid phase was discarded. The centrifuging process was repeated twice more after adding some amount of DI water to the solid phase and mixing together. Then, the colloidal precipitate Ni(OH)_2_ was dried at 80 °C for 6 h and calcined at 270 °C for 2 h to obtain NiO*_x_* NPs by the following reaction [54]:

Ni(OH)_2_ → NiO*_x_* + H_2_O (1)

The well-dispersed NiO*_x_* NP ink was prepared by dissolving NiO*_x_* NPs (23.8 wt%) and PVP (5.60 wt%) into 1-pentanol (70.6 wt%) using ultrasonication for 15 h.

Polyethylene terephthalate (PET, thickness ~25 µm) and PI (thickness ~50 µm) were cleaned by ethanol and used as substrates. The surface of the substrates was treated by oxygen plasma (BD-10A High-Frequency Generator, Chicago, IL, USA) to improve the adhesion between the substrates and the NP ink. Uniform NiO*_x_* thin films were prepared on both PET and PI by spin coating at 1000 rpm for 60 s and dried naturally under the ambient atmosphere for 30 min.

### 2.2. Laser Setup for the Laser Digital Patterning Process

**Figure 1:**
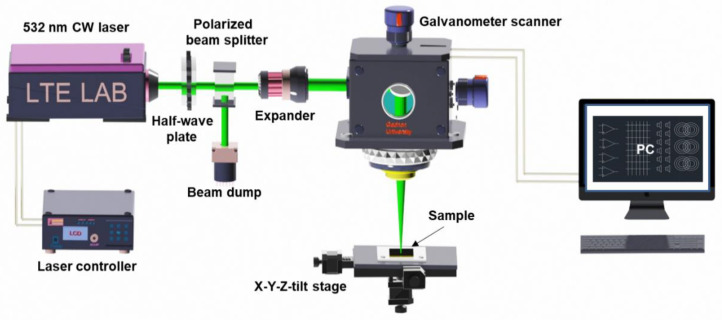
Schematic diagram of the laser setup for the laser digital patterning process to fabricate resistance temperature detectors.

A schematic illustration of the laser system setup for the LDP process using a 532 nm continuous wave (CW) Nd:YVO_4_ laser is illustrated in Figure 1. The focused laser beam irradiated the NiO*_x_* thin films through a galvanometer scanner (HurrySCAN III, Scanlab, Puchheim, Germany) consisting of scan mirrors and a telecentric f-theta lens (*f* = 100 mm). The half-wave plate and the polarized beam splitter were installed to control the laser power more precisely. The beam expander was used to enlarge the laser beam entering the telecentric lens installed in the galvanometer scanner to minimize the focused beam diameter. The diameter of the laser beam on the surface of the thin film was measured to be 25 µm. The laser power was precisely controlled by rotating the half-wave plate while the laser beam path for electrode patterning was controlled by the CAD system (laserDESK, Scanlab, Puchheim, Germany) that is linked to the scanner. The scanning speed was fixed at 50 mm s^−1^ and optimal laser power was found to produce the lowest resistance of a certain shape of the electrode. In this study, the laser power of 20 mW (power density: 4.1 kW cm^−2^) was applied to produce the RTD electrodes on both PET and PI substrates.

### 2.3. Characterizations

The sizes of NiO*_x_* NPs were estimated using transmission electron microscopy (TEM, JEOL JEM-2100F, Tokyo, Japan) images. The surface morphology of the sensor electrodes was characterized by scanning electron microscopy (SEM, Hitachi S-4800, Tokyo, Japan) and atomic force microscopy (AFM, Park System XE100, Suwon, Korea). Energy-dispersive X-ray spectrometry (EDS, Hitachi S-4800, Tokyo, Japan) analysis was conducted to investigate the elemental composition of the electrode. X-ray diffraction (XRD, Bruker D8 Advance, Billerica, MA, USA) patterns were recorded for phase identification. The resistance was measured using a multimeter (Agilent U1251B, Santa Clara, CA, USA) while the temperature was precisely controlled by a hot plate (Fisher Scientific, Hampton, NH, USA). The temperature of the hot plate also was monitored by a commercial thermocouple (Type K, EA11A). The resistivity (*ρ*) of the electrode was calculated using the equation: *ρ* = *R*·(*A*/*l*), where *R*, *A*, and *l* are the resistance, cross-sectional area, and length of the electrode, respectively.

## 3. Results and Discussion

**Figure 2:**
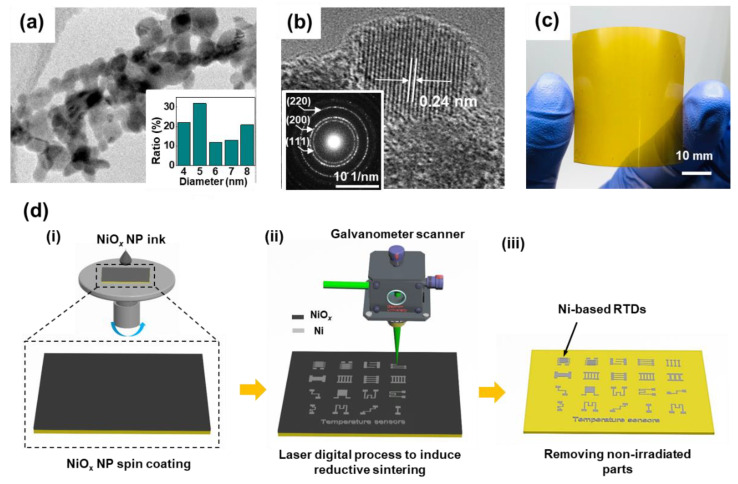
(**a**) Transmission electron microscopy (TEM) image of NiO*_x_* nanoparticles (NPs). The inset shows the size distribution of the synthesized NiO*_x_* NPs. (**b**) High-resolution TEM (HR-TEM) image of NiO*_x_* NPs. The inset is the selected-area electron diffraction (SAED) pattern for NiO*_x_* NPs. (**c**) A uniform NiO*_x_* NP thin film on a polyimide (PI) substrate. (**d**) Schematic diagram of the entire processes to produce Ni-based resistance temperature detectors (RTDs). (**i**) Spin-coating NiO*_x_* thin film on a polyimide substrate, (**ii**) laser digital process to fabricate Ni-based RTDs. (**iii**) Removing non-irradiated parts by the washing process.

The TEM image (Figure 2a) indicates that the sizes of NiO*_x_* NPs are in the range of 4–8 nm; their size distribution is displayed in Figure 2a inset. The high-resolution TEM image and the selected-area electron diffraction (SAED) pattern shown in Figure 2b and inset, respectively, demonstrate that NiO*_x_* NPs have a cubic crystalline structure with the distance of 0.24 nm between two successive bright fringes which corresponds to the (111) plane of NiO*_x_* [53]. Owing to the well-dispersed NP ink containing ultra-small NPs, smooth and uniform NiO*_x_* thin films can be coated on the substrate by spin-coating (Figure 2c).

The LDP process by which flexible Ni-based RTDs are produced by the LRS phenomenon is illustrated in Figure 2d. First, a NiO*_x_* thin film was deposited on the substrate by spin-coating of the NiO*_x_* NP ink. After drying the thin film under ambient atmosphere, the selective laser irradiation was applied on the NiO*_x_* thin film to produce Ni electrodes. The mechanism of the LRS phenomenon of PVP-containing metal oxide thin film has been reported in the previous studies [55,56], and can be applied to this work. In short, PVP incorporated in the NiO*_x_* NP thin film thermally decomposes upon laser irradiation and generates carboxylic acid that reduces NiO*_x_* to Ni. The reduced Ni NPs subsequently sinters together, and form a continuous conductive Ni electrode. Arbitrary patterns of Ni-based RTDs are easily fabricated on the substrate within a short time (Video S1 for the demonstration of the laser process). Last, the non-irradiated NiO*_x_* parts were easily washed away by rinsing with DI water or suitable solution, while the irradiated parts strongly adhered to the substrate (Video S2, Supporting Information). The LDP process applied on the solution-processed NiO*_x_* NP thin film to fabricate flexible Ni electrodes for RTDs offers the following advantages: (1) the entire processes from the NP synthesis to electrode patterning are conducted under ambient conditions without using any vacuum chamber or gas flow; (2) direct patterning without using a physical photomask is achievable and the shape of electrodes can be easily tuned; (3) thermally vulnerable thin flexible polymers can be employed as substrates since fast heating and cooling nature of localized laser heating minimizes thermal stress exerting on them.

**Figure 3:**
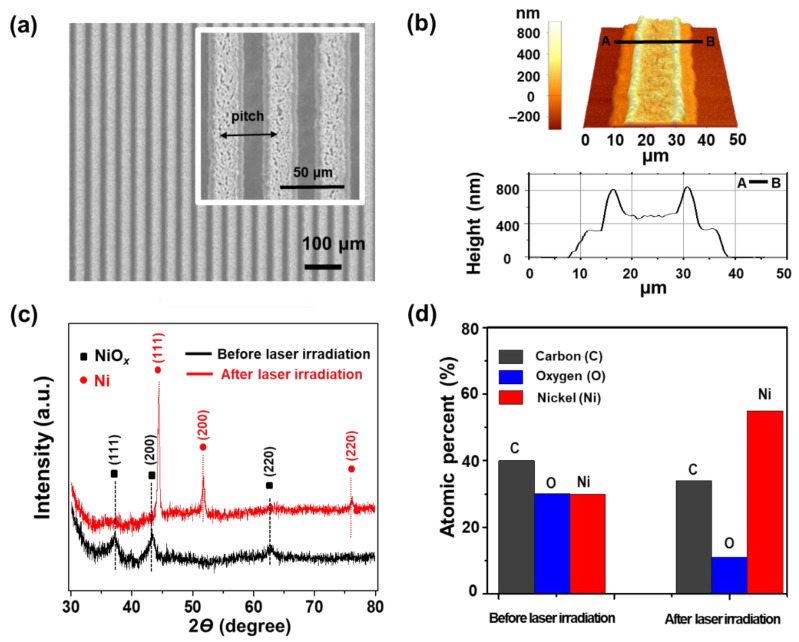
(**a**) Scanning electron microscopy (SEM) image of multiple Ni electrodes on the polyimide (PI) substrate. The inset is the SEM image at a higher magnification. (**b**) Atomic force microscopy (AFM) image of a single Ni electrode and the corresponding cross-sectional profile. (**c**) X-ray diffraction (XRD) patterns before and after the laser-induced reductive sintering of the NiO*_x_* nanoparticle (NP) thin film. (**d**) Comparison of a chemical composition detected by X-ray spectrometry (EDS) measurement before (left columns) and after (right columns) laser-induced reductive sintering of the NiO*_x_* NP thin film.

The surface morphology of Ni electrodes on the PI substrate fabricated by the LDP process at the laser power of 20 mW and scanning speed of 50 mm s^−1^ was characterized using SEM (Figure 3a) and AFM (Figure 3b). The width of the electrode (30 μm) is larger than the focused laser beam diameter (25 μm) due to heat diffusion across the thin film while the nominal thickness of the electrode is measured to be about 500 nm. It is worthwhile to note that the two axes for the cross-sectional height in Figure 3b have different scales. The phase change that occurred by the LRS phenomenon was confirmed by XRD data shown in Figure 3c. Before laser irradiation, the XRD pattern of the thin film shows peaks at 37.2°, 43.3°, and 63.2° corresponding to (111), (200), and (220) planes of the face-centered cubic (FCC) crystal structure of NiO*_x_*, respectively (JCPDS file no. 01-089-3493). After laser irradiation, the XRD pattern exhibits peaks at 44.4°, 51.7°, and 76.2° matching with (111), (200), and (220) planes of FCC Ni, respectively (JCPDS file card no. 01-087-0712). The reduction phenomenon was further confirmed by EDS data acquired with the NiO*_x_* thin film and the Ni electrode on the PI substrate as shown in Figure 3d. Ni content in the laser-irradiated region was higher than that in the non-irradiated region. The carbon peaks in both cases could come from the PI substrate and the PVP that exists in the thin film. Detection of oxygen element in the laser-irradiated region is possibly due to incomplete reduction of NiO*_x_* NPs and the formation of native oxide layers on the Ni electrode surface. The resistivity (*ρ*) of the Ni electrode is calculated to be 975 nΩ m which is about 14 times higher than that of bulk Ni (69.3 nΩ m).

**Figure 4:**
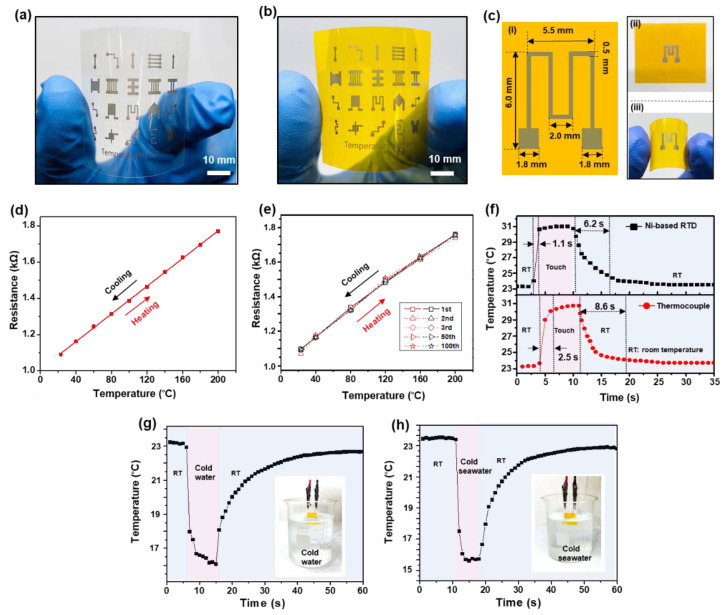
(**a**) Photo image of Ni-based resistance temperature detectors (RTDs) on a polyethylene terephthalate (PET) substrate. (**b**) Photo image of Ni-based RTDs on a polyimide (PI) substrate. (**c**) Schematic drawing of the Ni-based RTD (**i**) and photo images of the real sample (**ii** and **iii**). (**d**) The resistance variation with temperature showing a linear relationship. (**e**) 100 thermal cyclic tests of the Ni-based RTD. (**f**) Responses of the Ni-based RTD (**top**) and a commercial thermocouple (Type K, EA11A) (**bottom**) to the latex-gloved finger touching test. Responses of the Ni-based RTD to (**g**) tap water and (**h**) seawater. The insets are photo-images of the Ni-based RTD immersing in the water and seawater, respectively.

It is demonstrated that arbitrary Ni electrode patterns could be fabricated on an ultrathin (thickness: 25 µm) PET substrate that has a much lower glass transition temperature (~80 °C) than PI, as shown in Figure 4a. However, the performance of a Ni-based RTD on the PET substrate is limited due to the low glass transition temperature of the substrate. Therefore, the performance analysis of Ni-based RTDs in this study was executed using RTDs fabricated on a PI substrate which possesses high thermal stability [57]. Additionally, the thermal expansion coefficient of PI (~3 × 10^−5^ °C^−1^) [58] is close to that of Ni (~1.4 × 10^−5^ °C^−1^) [59]. Therefore, the possibility of any failure due to a large thermal expansion mismatch between the electrode and the substrate can be excluded. Figure 4b shows multiple Ni-based RTDs of various shapes. Areal Ni electrodes without vacant spaces between single Ni electrodes were generated by partly-overlapped parallel scanning of the laser beam with a pitch of 20 µm considering that the width of the single Ni electrode is 30 µm as mentioned above. It is worthwhile noting that no protective layer was added to the Ni-based RTD owing to its high thermal stability and strong adhesion on the substrate. The RTD performance of one Ni electrode sample that is selected among various samples shown in Figure 4b was investigated by measuring its electrical resistance variation from room temperature (RT = 23 °C) to 200 °C. The photo-image and the size detail of the selected sample are displayed in Figure 4c. The sample was placed on a temperature-controlled hot plate and its electrical resistance variation with temperature was recorded by a digital multimeter. For each measurement, the temperature of the hot plate was increased to the desired value and then maintained for 5 min to ensure a thermal equilibrium between the sample and the hot plate. The resistance of the sample increases from 1.09 kΩ at RT to 1.77 kΩ at 200 °C during the heating process and returns to its original value during cooling with almost no hysteresis (Figure 4d). More importantly, the relationship between temperature and resistance shows high linearity, which is desirable for an ideal sensor. Compared to the narrow temperature range of Cu-based RTDs due to oxidation issues, the Ni-based RTDs can operate without oxidation problems, which could be attributed to the oxidation-resistant property of the nickel itself and the native oxide layer formed on the electrode surface as discussed above. The temperature coefficient of resistance (*α*) is a common characteristic parameter that indicates the sensitivity of RTDs, and is described by the following equation [60]:

(2) $$\alpha =\frac{1}{R(T_{0})}\frac{R\left(T\right)-R\left(T_{0}\right)}{T-T_{0}}$$

where *R*(*T*_0_) is the resistance at a reference temperature *T*_0_ which is the RT in this study, and *R(T)* is the resistance at an elevated temperature *T*. The *α* value of Ni-based RTD is calculated to be about 3.52 × 10^−3^ °C^−1^, which is higher than those of Au- [36], Ag- [38], Cu-based RTD [44], and slightly smaller than that of the commercial platinum (Pt) temperature sensor (3.92 × 10^−3^ °C^−1^) [61]. To assess the reliability of the sensor performance, a thermal cyclic test was conducted by heating and cooling the sensor between RT and 200 °C for 100 cycles. The resistance variation from 1.09 kΩ at RT to 1.77 kΩ at 200 °C was maintained during the cyclic test (Figure 4e). The response speed of the RTD was evaluated by a latex-gloved finger touching test. The time of the resistance change resulting from the contact and isolation of the gloved finger on the RTD was recorded using a data logger software. Note that the Ag paste was applied on the two ends of the RTD and copper wires were used for the connection between the RTD and the multimeter. As the gloved finger contact the RTD, the resistance increased rapidly and was saturated after 4 s. Upon detaching the gloved finger from the RTD, the resistance returned to its original value after 8 s as shown in Figure 4f top and recorded in Video S3 in Supporting Information. In contrast, there was no change in resistance of the RTD in contact with objects of different shapes that were in thermal equilibrium with the room environment, which indicates that the effect of other parameters such as pressure force and contact area was much smaller than that of gloved-finger contact. The resistance variations during the gloved-finger test were converted to the temperature applying equation 2 with *α* = 3.52 × 10^−3^ °C^−1^ and equilibrium temperature was determined to be 31 °C. To compare the performance of the fabricated Ni-based RTD with that of a commercial thermocouple (Type K, EA11A), the same test was conducted using the commercial thermocouple, and the result is shown in Figure 4f bottom. The response times and the recovery times for the RTD, which is defined by the time taken by the signal change between a specified low threshold (10%) and a specified high threshold (90%), were indicated in Figure 4f. It is clear that the response and recovery times of the Ni-based RTD were faster than those of the commercial thermocouple, and the Ni-based RTD can precisely measure the temperature of the gloved finger. The fast response of the Ni-based RTD is attributed to the low thermal capacity of the thin substrate (50 μm) that allows fast reactions to temperature variations [62]. To demonstrate the high stability of the Ni-based RTD in various environments, the RTD was applied to measure the temperature of tap water and seawater, as shown in Figure 4g,h, respectively, (see Video S4 in Supporting Information).

**Figure 5:**
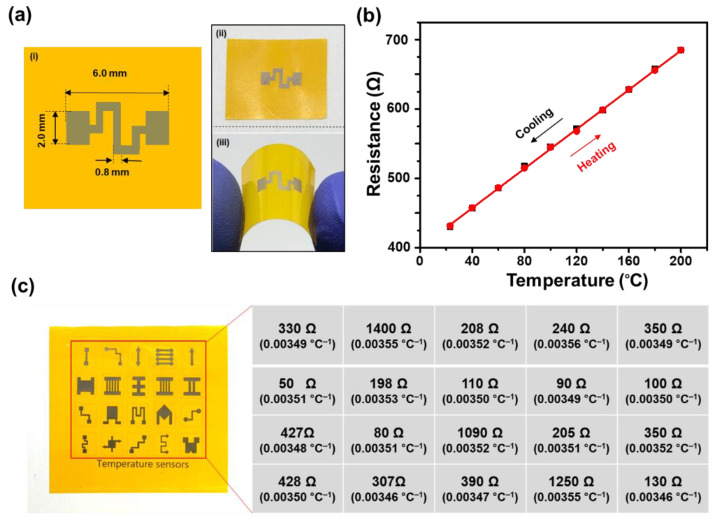
(**a**) Schematic drawing of the Ni-based resistance temperature detector (RTD) (**i**) and photo images of the real sample (**ii** and **iii**). (**b**) The resistance variation with temperature of showing a linear relationship. (**c**) The reference resistance values and the corresponding temperature coefficient of resistance (*α*) values of the Ni-based RTDs of various shapes. Each set of values corresponds to the RTD of the designated position.

To verify the effect of the electrode shape on the temperature-sensing performance, which affects the reference resistance of the electrode, the Ni-based RTD of another shape was selected and the *α* value of it was examined. The schematic drawing and photo images of the real sample are shown in Figure 5a. The temperature–resistance relationship of the RTD was measure in the same manner and verified that the resistance of the sample changes linearly from 428 Ω at RT to 693 Ω at 200 °C (Figure 5b) during heating and cooling without hysteresis. It is noted that even though the room temperature resistance (428 Ω) is different from that of the first sample (1.09 kΩ) because the dimension of the electrode is different, the *α* value of the sample (3.50 × 10^−3^ °C^−1^) is almost same as that of the first RTD sample (3.52 × 10^−3^ °C^−1^). The room temperature resistance and corresponding *α* value of each RTD of different shapes are summarized in Figure 5c. The variation of the *α* values are within 2.6% regardless of the room temperature resistance of the RTD. This result shows that the sensitivity of the Ni-based RTD does not depend on the extensive properties of the Ni electrodes, which provides convenience in practical use by offering design pliability.

**Figure 6:**
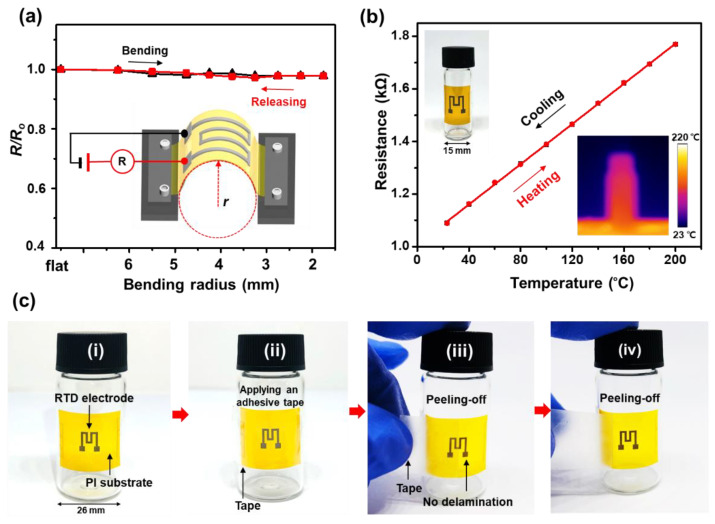
(**a**) Relative resistance changes of the Ni-based resistance temperature detector (RTD) under various bending radii. (**b**) Resistance variation with temperature under the bending condition. (**c**) Tape-pull test of the Ni-based RTD on the PI substrate. (**i**) RTD attached on the surface of a 26-mm-diameter glass vial, (**ii**) applying tape (Scotch^®^ Magic^TM^ tape, 3M) on top of the electrode, (**iii**,**iv**) repeating peeling off the tape.

Mechanical robustness is also an important requirement for flexible RTDs. To examine the mechanical stability of the RTDs, the resistance variation (*R/R_0_*) under various bending radii was measured, where *R_0_* is the initial resistance and *R* is the resistance under the bending condition. The resultant resistance variation (*R/R_0_*) is less than 3% up to a bending radius of 1.75 mm indicating a high-level stability under mechanical deformation (Figure 6a). The small resistance variation indicates that there is no permanent damage to the electrodes under bending conditions. The high electrical reliability could be attributed to the lowered bending stress owing to the thin thickness of the sensor (~50 µm) as well as the fully-densified microstructure and robust adhesion of the electrode on the substrate. It should be noted, however, that 3% of the resistance variation corresponds to 8.5 °C temperature difference based on the given α = 3.52 × 10^−3^ °C^−1^, which is not negligible. Therefore, if the RTD is attached to a surface where the radius of curvature of the surface actively varies over a wide range from ∞ (flat) to 1.75 mm, the error in the measured temperature can be that large, which means that the Ni-based RTD on polyimide has some limitation to be applied to such a surface. However, if the curvature variation decreases, the measurement error can be reduced. For example, if the radius of curvature of the surface varies in the limited range such as flat (∞) ~6.5 mm, and 5 mm ~ 3 mm, the errors in the measured temperature are less than 1 °C and 2 °C, respectively. The mechanical robustness of the Ni-based RTD suggests that the RTD can be applied to curved surfaces without degrading its performance. For instance, the performance of the Ni-based RTD under the bending condition was evaluated by attaching the sensor to the curved surface of a 15-mm-diameter glass vial as shown in the left inset of Figure 6b. The vial was filled with 4 mL of silicon oil to ensure the uniform heat distribution on the curved surface, then placed on a hot plate to increase the temperature. The right inset of Figure 6b confirms the uniform surface temperature during heating. The resultant resistance variation with temperature did not change when compared to the change in resistance of the specimen in a flat state (Figure 6b). The adhesion of Ni-based RTDs on the PI substrate was investigated by carrying out a tape-pull test. The tape-pull test was conducted by applying conventional adhesive tape (Scotch^®^ Magic^TM^, 3M) to the electrode surface and peeling it off subsequently several times. It was confirmed that the Ni-based RTDs did not detach from PI as shown in Figure 6c (see Video S5 in Supporting Information). The strong adhesion of Ni-based RTDs on the PI substrate is attributed to the melting-solidification process that happens at the interface of the electrode and the substrate resulting in interlocking of them as discussed in our previous study [53].

## 4. Conclusions

In summary, we have demonstrated a facile and rapid LDP process to fabricate low-cost, Ni-based flexible RTDs. Ultrasmall NiO*_x_* NPs were synthesized by a scalable chemical precipitation method, and smooth and uniform thin films could be spin-coated owing to the well-dispersed NP ink. The LRS phenomenon facilitated the generation of various shapes of Ni electrodes on a thin flexible substrate using the NiO*_x_* thin film without using a physical photomask. The entire processes from the NP synthesis to the laser process were operated under ambient conditions without involving any vacuum process. The flexible Ni-based RTDs exhibited fast response and excellent repeatability in a wide temperature range up to 200 °C. The temperature coefficient of resistance, or sensitivity of the RTD (3.52 × 10^−3^ °C^−1^) was higher than those of Au-, Ag-, Cu-based RTDs reported in previous literature and was comparable to that of the commercial Pt-based one. Moreover, the sensitivity of the Ni-based RTDs was independent of the electrode shape and the reference resistance, which is advantageous in practical application by offering design flexibility. The bending test and the tape-pull test confirmed the superior mechanical and electrical stability of the flexible RTDs. This novel yet simple LDP process for the generation of the Ni-based flexible RTDs offers a new way to replace conventional fabrication methods and materials.

## Supplementary Materials

The following are available online at https://www.mdpi.com/2079-4991/11/3/576/s1, Video S1: Laser digital patterning processing, Video S2: Washing process, Video S3: Response to gloved-finger touching, Video S4: Immerse in cold tap water and seawater, Video S5: Tape-pull test.
